# The diagnostic and prognostic potential of gut bacteria in inflammatory bowel disease

**DOI:** 10.1080/19490976.2023.2176118

**Published:** 2023-02-16

**Authors:** Dickson Kofi Wiredu Ocansey, Sanhua Hang, Xinyi Yuan, Hua Qian, Mengjiao Zhou, Chinasa Valerie Olovo, Xu Zhang, Fei Mao

**Affiliations:** a Key Laboratory of Medical Science and Laboratory Medicine of Jiangsu Province, School of Medicine, Jiangsu University, Zhenjiang, P.R. China; b Directorate of University Health Services, University of Cape Coast, PMB, Cape Coast, Ghana; c The People’s Hospital of Danyang, Affiliated Danyang Hospital of Nantong University, Zhenjiang, P.R. China; d Affiliated Hospital of Jiangsu University, Jiangsu University, Zhenjiang, P.R. China; e Department of Microbiology, University of Nigeria, Nsukka, Nigeria

**Keywords:** Gut bacteria, diagnosis, prognosis, inflammatory bowel disease, gastrointestinal disease, microbiota

## Abstract

The gut microbiome serves as a signaling hub that integrates environmental inputs with genetic and immune signals to influence the host’s metabolism and immunity. Gut bacteria are intricately connected with human health and disease state, with specific bacteria species driving the characteristic dysbiosis found in gastrointestinal conditions such as inflammatory bowel disease (IBD); thus, gut bacteria changes could be harnessed to improve IBD diagnosis, prognosis, and treatment. The advancement in next-generation sequencing techniques such as 16S rRNA and whole-genome shotgun sequencing has allowed the exploration of the complexity of the gut microbial ecosystem with high resolution. Current microbiome data is promising and appears to perform better in some studies than the currently used fecal inflammation biomarker, calprotectin, in predicting IBD from healthy controls and irritable bowel syndrome (IBS). This study reviews current data on the differential potential of gut bacteria within IBD cohorts, and between IBD and other gastrointestinal diseases.

## Introduction

Recent progress in our ability to characterize the gut microbiota has resulted in tremendous interest in identifying organisms associated with different human diseases, including gastrointestinal perturbations such as IBD. The human gut contains an abundant and diverse microbial community of trillions of microorganisms, including bacteria, viruses, and yeast. The gut microbiota, consisting mainly of bacteria species, plays a key role in human health, including the education and maturation of host immune responses, prevention of enteric pathogen proliferation, and response to or modification of certain medications.^1,2^ At the cellular level, host physiology can be altered by microbiome-induced cell signaling, proliferation, and neurotransmitter biosynthesis, resulting in mucosal and systemic changes, which consequently affect homeostasis, innate, and adaptive immune responses, intestinal barrier function, and metabolism.^3–5^ Moreover, microbial metabolism helps in the establishment and maintenance of health by producing metabolites from dietary substrates, and modification of host molecules, such as bile acids, or directly from bacteria.^6^ Signals from these microbial metabolites modulate host energy metabolism, maintain mucosal integrity, and assist in immune maturation and homeostasis.^7,8^ These observations indicate that gut bacteria are intricately connected with human health and disease state and thus, could be harnessed to improve disease diagnosis, prognosis, and treatment.

Although the etiology of IBD remains unclear, it is well accepted that the intestinal microbiota is associated with not only the development but maintenance of IBD.^9^ Several studies have reported that gut bacteria play a critical role in triggering and maintaining the inflammatory process in the gut tissues of IBD patients by supplying antigens or other stimulatory factors that initiate immune cell activation.^10,11^ Significant alteration is reported across the four major bacterial phyla, Firmicutes, Bacteroidetes, Proteobacteria, and Actinobacteria, which together constitute >98% of the gut microbiota.^12,13^ Specific bacteria with a strong correlation with gut dysbiosis include *Shigella/Escherichia, Faecalibacter-ium prausnitzii, Alistipes, Bifidobacterium, Dialister, Eubacterium, Akkermansia, Lactobac-illus/Pediococcus, Bacteroides fragilis, Ruminococcus albus/bromii, Ruminococcus gnavus*, and *Streptococcus sanguinis/thermophilus*.^11,14–16^ This makes the gut microbiota an important factor in the pathogenesis of IBD and a potential diagnostic and prognostic biomarker. In an assessment of the gut microbiota as a diagnostic tool, a machine learning technique based on generalized linear models with penalized maximum likelihoods was applied, where the microbial composition showed a better IBD/IBS predictive accuracy [area under the curve (AUC)_mean_ of 0.91 (0.81 to 0.99)] than the currently used fecal inflammation biomarker calprotectin [AUC_mean_ of 0.80 (0.71 to 0.88); P = 0.002. Further analysis using the top 20 taxonomies with the largest effect size in the prediction model produced an AUC_mean_ of 0.90, while the top five taxonomies also gave a similar predictive accuracy as fecal calprotectin measurements (top five taxa, AUC_mean_ of 0.81, and AUC_calprotectin_ of 0.80).^11^ In a similar study in pediatric IBD, the combined application of 16S rRNA and shotgun sequencing could predict pediatric UC status with the area under the receiver operating characteristic curve (AUROC) of approximately 0.90 based on cross-validation.^17^ Another gut microbiota disease-predictive capability study reported that the overall IBD prediction was AUC 0.802, UC prediction of AUC 0.92, and CD prediction of AUC 0.863, concluding that microbiome indicators outperform human genetics in predicting host phenotype.^18^

Due to these interesting observations, an increasing number of researchers are exploring the observed differences in gut microbiome between IBD and healthy subjects, within IBD subtypes, and between IBD and other gastrointestinal inflammatory conditions through microbial taxonomy markers as potential differential predictors. In recent years, different microbial signatures have been reported to be specific in IBD states (active and remission), subtypes (CD and UC), and other gastrointestinal conditions. We summarize these studies in the context of the diagnostic and prognostic potentials and ways of maximizing outcomes in future studies.

## The contribution of gut bacteria to IBD pathogenesis

**Figure 1.**
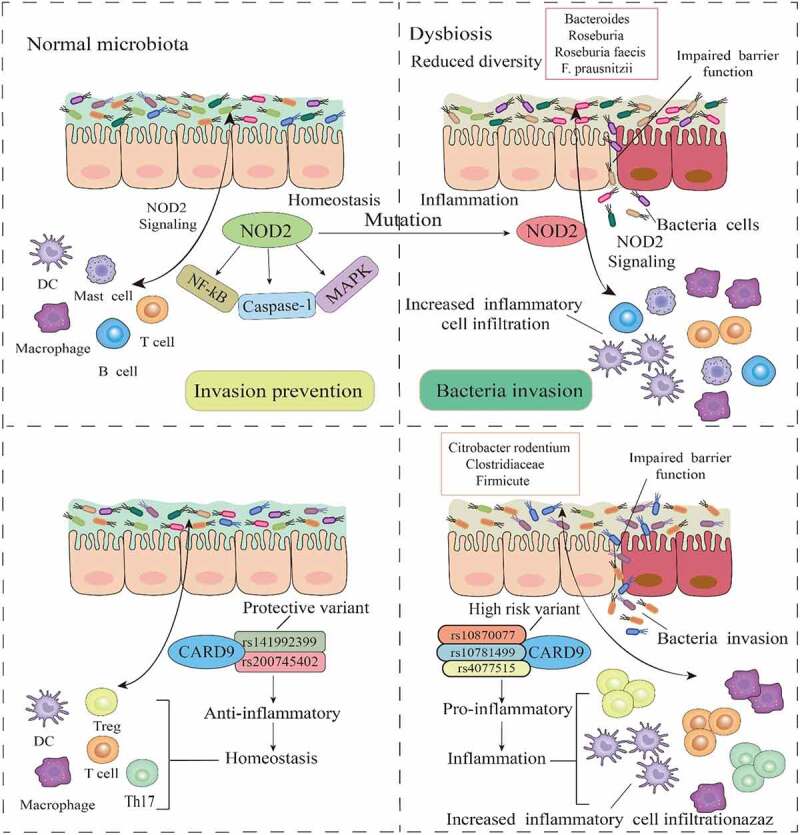
Gut bacteria-associated gene mutations in IBD. In a normal intestinal environment, the gut microbiota interacts with immune components via NOD2 to prevent infection. However, mutations in NOD2, which invariably affect the MAPK, NF-kβ, and caspase-1 pathways, influence microbial response, and promote low diversity and dysbiosis in the microbiome, leading to an impaired mucosal barrier function and bacteria invasion. As a key regulator of microbiota in the intestine, the dysfunction of NOD2 signaling correlates with certain gut bacteria. Mutations in CARD9 genes are also associated with certain gut bacteria, where mutation into high-risk and proinflammatory variants drive dysbiosis, impair barrier function, and increase inflammatory cells and cytokines.

In recent years, our understanding of the function and composition of the gut microbiota has tremendously increased, mainly due to the introduction of new ‘omic’ technologies that have produced large-scale analyses of the metagenomics and metabolomics profile of the microbial community, revealing a comparable in influence to a ‘new organ’ in the body and providing the potential of a new diagnostic marker and therapeutic route.^5,19^ Early studies that investigated the role of gut bacteria in the pathogenesis of IBD aimed at identifying potential bacteria that could trigger the inflammatory cascade typical of IBD. Several culprits have been proposed including *Clostridium difficile*,^20^ *Mycobacterium avium subsp paratuberculosis*,^21,22^ and a number of Proteobacteria such as enterohepatic *Helicobacter, non-*jejuni/coli *Campylobacter*,^23–25^ and adherent and invasive *Escherichia coli*.^26,27^ However, the focus has recently been broadened with the realization that the gut microbiota as a whole is altered in IBD. The participation of host-gut microbe interactions in the pathogenesis of IBD continues to be highlighted by metagenomics, metabolomics, and genome-wide association analyses of fecal or biopsy samples. Earlier genetics research that recruited an extended cohort of 86,640 patients with IBD and control, implicated 38 loci in IBD risk, where mutations of some of the genes often affect specific host pathways associated with microbial response in IBD, such as the NOD2-mediated innate immune response to bacterial infections, the interleukin (IL)-23 pathway, autophagy, and Paneth cell functions.^28,29^ Six bacteria groups (Bacteroidia, Bacteroidaceae, Bacteroides, Roseburia, *Roseburia faecis, and Faecalibacterium prausnitzii*) were identified to be involved in NOD2 signaling, while one (Firmicutes) was involved with CARD9.^30^ CARD9 alleles in IBD patients have both a common predisposing risk and rare protective function and are linked with gut bacteria such as *Citrobacter rodentium*, Firmicutes, and Clostridiaceae. The CARD9 variants rs4077515, rs10870077, and rs10781499 are confirmed to be high genetic risk factors, whereas rs200735402 and rs141992399 are shown to have a functional protective role.^31^ These results indicate that gut bacteria are not only implicated in the induction of the characteristic dysbiosis observed in IBD but host genetic mutations that drive and sustain the disease (Figure 1).

**Figure 2.**
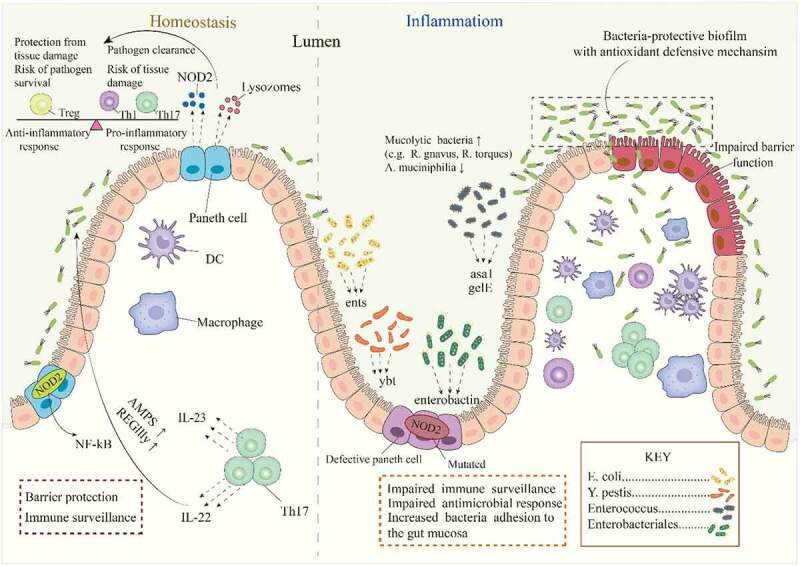
The contribution of gut bacterial virulence factors and mucolytic bacteria to IBD pathogenesis. In the intestinal mucosa, Paneth cell secretes NOD2 and lysozyme to maintain a balance of pro-inflammatory and anti-inflammatory immune mediators, and control the population of certain mucolytic bacteria. The characteristic dysbiosis in IBD is linked with dysfunctional Paneth cells, increased bacterial populations that produce virulence factors, increased bacteria adhesion, and the formation of bacteria biofilms with defensive mechanisms. These factors further impair immune response and drive inflammation, leading to a defective barrier function and bacteria invasion.

At the phylum level, some of the most consistently reported taxonomic shifts in IBD include the depletion of Firmicutes and Bacteroides and elevated abundance of Proteobacteria and Actinobacteria.^15^ Moreover, epithelial-associated bacteria of the large and small intestine are more likely to be direct key players in the etiology of IBD due to their more direct interaction with the affected tissues and the mucosal immune system, whereas fecal microbiota presents significant microbial alterations that characterize the dysbiosis observed in IBD.^32,33^ Bacteria virulence factors contribute to pathogenic potential in IBD through several mechanisms, including immune system evasion or suppression of the host immune response and increased adhesion of bacteria to the gut mucosa. For example, the relative abundance of 262 virulence factors was found to increase in IBD and IBS patients compared with controls, including entS, usually found in *E. coli*, yersiniabactins (ybt), mostly found in *Yersinia pestis*, and enterobactins, which correlated with the relative abundance of Enterobacteriales.^11^ Similarly, Golińska and collogues reported increased frequency in virulence factors [surface aggregating protein (asa1) and gelatinase (gelE)] of *Enterococcus* strains isolated from patients with IBD compared with healthy controls.^34^ The authors concluded that *Enterococcus* strains that adhere strongly to the intestinal epithelium, form biofilms and possess antioxidant defense mechanisms that seem to have the greatest influence on the inflammatory process. Although mucolytic bacteria are present in the gut of healthy humans where they function as an integral part of the mucosa-associated bacterial consortium, they are reported to increase in IBD.^16,35,36^ A study found mucolytic bacteria to increase by an average of 1.9-fold in CD and 1.3-fold in UC, with specific bacteria such as *Ruminococcus gnavus* and *Ruminococcus torques* increasing by >4-fold and ∼100-fold, respectively. Interestingly, the most abundantly detected mucolytic bacterium in healthy controls, *Akkermansia muciniphila*, decreased several folds in both CD and in UC.^16^ The disproportionate elevation in the abundance of mucolytic bacteria such as *Ruminococcus gnavus* and *R. torques* may contribute to the pathogenesis of IBD by providing substrate to sustain non-mucolytic mucosa-associated bacteria and thus, explain the increased total mucosa-associated bacteria in IBD. In addition, distortions in Paneth cell secretions (decreased lysozyme), which is known to balance intestinal anti- and pro-inflammatory responses, result in the expansion of lysozyme-sensitive mucolytic bacteria such as *Ruminococcus gnavus*,^37^ with implications for IBD pathogenesis (Figure 2).

It is worth noting that in addition to gut bacteria, other microorganisms in the microbiota such as fungi and viruses may also contribute to the pathogenesis of IBD. Compared with healthy individuals, fungal microbiota is significantly altered in IBD, with an elevated Basidiomycota/Ascomycota ratio, reduced abundance of *Saccharomyces cerevisiae* and an increased proportion of *Candida albicans*. Further analysis led to the identification of a distinct fungal microbiota dysbiosis in IBD characterized by changes in composition and biodiversity.^38^ Another study found that Basidiomycota and Ascomycota phyla dominate the mucosa of CD patients, with overexpression of Cystofilobasidiaceae family and *Candida glabrata* species. Moreover, *Saccharomyces cerevisiae* and *Filobasidium uniguttulatum* species were associated with non-inflamed mucosa, whereas Xylariales order was associated with inflamed mucosa.^39^ Concerning the gut virome, researchers have observed IBD-specific changes to the virome and elevated abundance of temperate phage sequences in CD patients, where the alterations in virome composition reflected changes in bacterial composition. However, the incorporation of both bacteriome and virome composition produced a greater classification power in distinguishing IBD patients from healthy individuals.^40^

## Sources of samples

### Fecal

In recent years, new techniques have permitted researchers to phylogenetically identify and quantify the constituents of the gut microbiota by analyzing RNA and DNA directly extracted from fecal samples. The majority of these technologies are based on the extraction of DNA and the amplification of the 16S ribosomal RNA gene (rRNA) as the most useful sequencing technique to highlight the diversity and abundance of the microbiome.^41,42^ Fecal matter presents a convenient and repeatable sampling that is noninvasive, inexpensive, and offers sufficient biomass for analysis. Fecal samples are the most commonly used gut microbiota samples and are reported to offer sufficient bio-information to differentiate patients from healthy individuals across several conditions, including IBD,^43,44^ colorectal cancer,^45,46^ neurodegenerative diseases,^47,48^ cardiovascular diseases,^49^ and metabolic diseases.^50^ However, it is confronted with challenges such as the inability to accurately reveal the changes in gut microbiota, partly due to the uneven distribution of bacteria. It was found that 35% of low-abundance taxa of the total microbiome in one replicate, were not found in a second fecal sample.^51^ Other reports indicated that the fecal- and mucosal-associated microbiota are two distinct microbial niches, and fecal samples could not serve as an accurate indicator of the compositional and metagenomic function of mucosa-associated microbiota distributed within multiple sites of the intestine.^52,53^ While the families *Bacteroidaceae, Prevotellaceae, Ruminococcaceae, Rikenellaceae*, and *Lachnospiraceae* are dominant in the colon, the families *Enterobacteriaceae* and *Lactobacillaceae* are rather predominant in the small intestine,^54^ thus, fecal samples may only be representative and may present an inadequate picture of the state of the gut. Regardless of these defects, fecal samples remain the most frequently used in gut microbial analysis and offer a lot of potential in diagnostic and therapeutic assessment.

### Tissue biopsy

**Figure 3.**
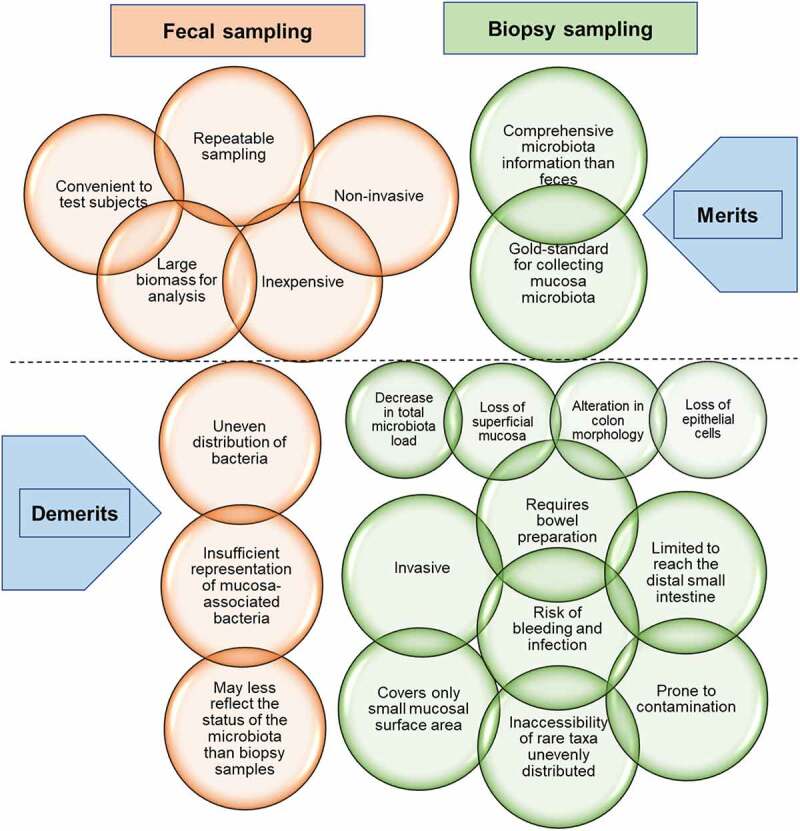
Advantages and disadvantages of fecal and biopsy sampling methods for microbiota analysis. Although fecal samples present sufficient bio-information and are the most commonly used for microbiota analysis, intestinal biopsies offer more comprehensive microbiota information. Regardless, fecal sampling remains the most frequent due to the many disadvantages associated with biopsy sampling. Note: The upper brown circles represent the merits of fecal sampling while the lower ones represent the demerits. The upper green circles represent the merits of biopsy sampling while the lower ones represent the demerits. The four green smaller circles are the consequences of bowel preparation for biopsy sampling.

Compared with fecal samples, fewer studies have utilized tissue biopsy and luminal contents samples to assess the gut microbiota in different microbial niches. Such samples are obtained through endoscopic procedures via the use of tools such as biopsy forceps and luminal brushes and could offer more comprehensive information on the gut microbiome than fecal samples. For example, a study of treatment naïve CD patients found that several taxonomic biomarkers measured at both the ileal and rectal sites using biopsies, significantly correlated with the disease phenotype; however, most of that microbial signal was lost in the stool samples.^55^ Nonetheless, tissue biopsy sampling is invasive, requires bowel preparation, difficult for patients, and is limited to reaching the distal small intestine. Bowel preparation with laxatives significantly changes gut microbiota composition and diversity, and significantly causes morphological alterations in the colon, including the loss of superficial mucus and epithelial cells.^56–58^ Moreover, lavage before colonoscopy results in a 31-fold decrease in the total microbial load and the loss of the subject specificity of the microbiota of patients.^59^ Tissue biopsy sampling is again prone to contamination during the procedure, only covers a small mucosal surface area, and may result in sampling deviation and inaccessibility of rare taxa unevenly distributed. Again, co-purification of large amounts of host DNA makes metagenomics challenging in biopsy sampling. Although tissue biopsy is deemed the gold standard for the collection of mucosal microbiota, the above challenges coupled with the risk of bleeding and infection make it largely inappropriate for gut microbiota analysis, thus, infrequently used. The merits and demerits of fecal and biopsy sampling methods are shown in Figure 3.

## Key analytical techniques

In the past, gut microbiota analysis was carried out through culture and isolation, but the inability to culture certain microbes coupled with the difficulty in cultivating anaerobic bacteria, which are abundant in the gut, contributed to the development of next-generation sequencing techniques. These techniques can accurately analyze gut microbial components down to the species- and strain-level from sample sources such as feces, mucosal biopsy, and intestinal aspiration. Culture-independent and -dependent approaches have both been employed in gut microbial community analysis to assess the involvement of microorganisms in different diseases, including IBD. Culture-independent techniques, including next-generation sequencing methods such as 16S rDNA gene sequencing and whole-genome shotgun sequencing, have proven much more reliable and faster in profiling complex microbial communities. The next-generation sequencing methods are based on sequence divergences of the small subunit ribosomal RNA (16S rRNA), considered the universal target for bacterial identification from DNA, or other target gene regions.^60,61^ Other non-next-generation sequencing methods include quantitative real-time PCR (qPCR), fluorescence in situ hybridization (FISH), terminal restriction fragment length polymorphism (T-RFLP), and denaturing gradient gel electrophoresis (DGGE), and DNA microarrays.^62^

Next-generation sequencing is the current method of choice to characterize microbial community composition and 16S rRNA gene (rDNA) amplicon analysis remains the standard approach for the cultivation-independent investigation of microbial diversity.^63,64^ In addition to the 16S rRNA gene sequencing technique, shotgun metagenomic sequencing has also been used to investigate the contribution of the gut microbiota and its associated pathways to IBD.^1,11^ Shotgun metagenomics data, along with metabolomics can assist in the exploration of the various mechanisms by which the human gut microbiota participates in the onset and development of IBD. It was shown that both shotgun sequence data and metabolite could predict disease status with high AUROC between 0.86 and 0.92.^10^ To complement data obtained from the 16S rRNA sequencing, shotgun metagenomics analysis is usually performed, a technical approach that provides elevated resolution, enabling a more specific taxonomic classification of sequences and giving a direct measurement of the functional characteristics of the gut microbiome.^65^ However, when each technique was used alone, it was discovered that compared to 16S rDNA amplicon sequencing, shotgun next-generation sequencing metagenomics allows a much deeper characterization of the microbiome complexity, allowing the identification of a larger number of species for each sample used.^66^ Regardless, both technologies use massive gene sequencing to generate sufficient bioinformatic data that have resulted in important progress in the human microbiome, achieving an unprecedented detail level on the taxonomy and microbial function.

## Levels of gut bacteria alteration

The crosstalk between the microbiota, gut epithelium, and the gut immune system determines the individual health status, and any disturbance in this interaction may result in chronic intestinal inflammatory conditions such as IBD. To this end, alterations in the composition and function of the gut microbiota have been documented in several studies on IBD including alterations in the metabolite profiles and their associated pathways in the same patients.^43,67^ In metabolomics analysis, specific classes of metabolites, notably bile acids, short-chain fatty acids, and tryptophan have been implicated in the pathogenesis of IBD.^6^ This also means that well-integrated correlation analysis between gut bacteria, metabolites, and related functions and pathways in large studies could produce better diagnostic and prognostic tools.

### Alteration in bacterial diversity

The pathogenesis of IBD is not only linked with changes in the composition of the intestinal microbiota but also a reduced diversity of intestinal microbial species. A measure of diversity, which refers to the richness of individual bacteria from each of the bacterial species present in the gut microbiome, offers a differential marker for IBD. While many previous reports indicate that species richness in the gut of both CD and UC patients is lower than that of healthy subjects,^68,69^ others further indicate that CD patients exhibit substantially lower species richness in the gut than UC patients.^12^ Moreover, a recent study found that whereas IBD patients had lower intestinal bacterial diversity, CRC patients rather had higher diversity compared to healthy subjects.^70^ Further studies are needed to confirm this observation. Pediatric IBD is equally characterized by reduced species richness. For example, the α-diversity of pediatric UC patients is lower than that of healthy controls, whereas β-diversity between UC and healthy subjects is much higher than that between healthy samples, and correlated significantly with disease status.^17^ This observation is recently confirmed in adult IBD samples that revealed significantly reduced α-diversity [Chao 1, ACE, goods coverage, observed specifications] and increased β-diversity compared to healthy controls.^43^

### Alterations in the general community structure

The human microbiome is a complex and dynamic ecosystem, and the imbalance of the general microbial community structure, termed dysbiosis, characterizes intestinal inflammation. Studies show that approximately 99% of the gut microbiota, consisting of Firmicutes, Proteobacteria, Bacteroidetes, and Actinobacteria is perturbed in the IBD environment,^71,72^ with both IBD and healthy controls retaining unique OTUs.^43^ This indicates that the entire microbial community structure is altered in IBD patients, resulting in the emergence of unique changes that could offer differential application in diagnosis, prognosis, and treatment. Generally, OTUs are significantly reduced in both UC and CD.^43,73,74^ By mapping IBD-altered bacterial families to a network of the gut microbiome of >22,000 fecal and gut biopsies samples obtained from both diseased and healthy individuals, Alam and colleagues showed that the bacterial families which increase in relative abundance in IBD patients are not well connected to other groups, indicating that those bacteria families generally do not coexist together with common gut organisms. On the other hand, the bacterial families with reduced or nonsignificant change in relative abundance in IBD patients relative to healthy subjects are very well connected to other gut bacterial groups, implying that they are highly crucial bacteria groups in the gut that can coexist with other bacteria across a range of conditions.^12^ This is an important observation affirming IBD-specific gut bacteria increases, thus, the potential of gut bacteria in the diagnosis and prognosis of IBD and other gut inflammatory conditions.

### Alteration in microbial community stability and associated pathways

Normal microbial communities exhibit relative stability via functional resistance and resilience to prevent general environmental disturbances.^75^ Moreover, positive equilibria remain stable in a complex microbial community such as the gut, whenever mutualistic interactions are either sufficiently weak or when all pairs of taxa reciprocate each other’s assistance.^76^ However, the IBD environment exhibits a strongly dysregulated interaction between microbial, immune, and environmental factors, thus, producing impaired microbial community stability.^77^ IBD patients do not only suffer severe perturbation of gut bacteria community composition, diversity, and associated functions but metabolites and metabolic pathways. This invariably reduces microbial community stability. Some of the significantly affected pathways in the IBD cohort included primary bile acid biosynthesis, vitamin digestion and absorption, and carbohydrate metabolism.^43^ It is also observed that the pathway components unique to bacteria species that are reduced in CD, contribute to the bile acid and amino acid biosynthesis pathways, including connections between amino acid metabolism and energy, carbohydrate, or nucleotide metabolism,^55^ which together provide access to complex carbohydrates and a link to the TCA cycle,^78^ indicative of a true anaerobic bacteria lifestyle. This implies that anaerobic bacteria are reduced relative to aerotolerant bacteria, with an expansion of facultative anaerobic bacteria of the family *Enterobacteriaceae*. This is also reported by a study that observed that aerotolerant taxa such as Actinobacteria, Proteobacteria, and Fusobacteria were increased in IBD. In the same study, SCFA-producing bacteria such as *Butyricimonas* and *Odoribacter* were significantly decreased in both UC and CD patients.^79^ Moreover, IBD patients suffer reduced levels of other SCFA-producing bacteria like *Clostridium* and *Faecalibacterium prausnitzii* and an elevated abundance of mucolytic bacteria that degrade the mucus layer like *Ruminococcus gnavus* and *Ruminococcus torques*.^80^ Heme biosynthesis, L-alanine biosynthesis, and oleate biosynthesis are elevated in patients with UC,^17^ as demonstrated by others to the effect that dietary heme significantly changed the microbial composition, characterized by a decrease in α-diversity, a reduction of Firmicutes, and an increase of Proteobacteria, which cause marked gut dysbiosis, worsened colitis, and potentiated development of adenomas in mice.^81^ Moreover, serum fatty acids such as oleic acid correlate with inflammatory cytokines in patients with UC, implying that IBD subjects with increased oleic acid intake could alter the inflammation severity.^82^

### Alteration in the abundance of specific taxa and strain-level

**Figure 4.**
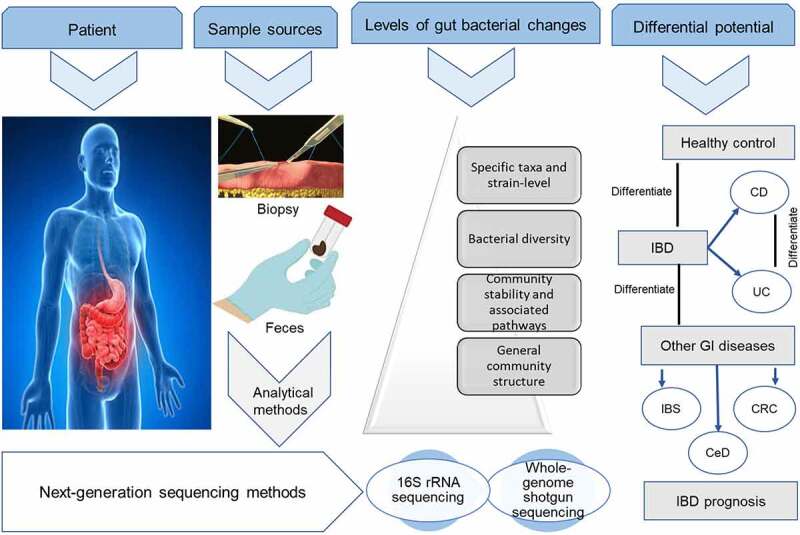
Sample sources and levels of gut bacteria changes. Alterations in the gut bacteria community can be assessed using next-generation sequencing techniques on fecal and biopsy samples to depict the changes at different levels of microbiota information. The specific taxa and strain-level information are the sharpest in differentiating IBD from healthy controls and other gastrointestinal diseases.

The observed changes of a general reduction in the OTU structure in the gut microbiome of both UC and CD patients are further expanded into variable alterations from the phylum to species levels, relative to a healthy gut microbiome.^70,83^ Fecal microbiota whole-genome sequencing confirms the presence of classic gut dysbiotic features of not only increased abundance of Proteobacteria, but also Actinobacteria and Fusobacteria, and decreased abundance of Firmicutes, Bacteroidetes, and Verrucomicrobia.^79^ At the family level, both 16S rRNA and shotgun data indicate that species from *Akkermansiaceae, Clostridiaceae, Eggerthellaceae, Lachnospiraceae*, and *Oscillospiraceae* are significantly depleted in UC patients compared with controls.^17^ These taxa changes are replicated through the entire phylogenetic tree of gut bacteria down to the specie and strain level. Species and strain-level identification of the gut microbiota are significant to the identification of potential disease-associated microbes that could be cultured and help investigate functional studies. Several studies have reported increased strain diversity of likely pathogenic species and reduced strain diversity in beneficial species in stool samples from IBD patients compared with controls. One such study found that the strain diversity of 21 bacterial species was altered in CD and 15 in UC compared with healthy controls.^11^ Although there are variable observations of specific species in IBD, two different studies ^11,17^ consistently reported a reduced abundance of the species *Adlercreutzia equolifaciens* and *Akkermansia muciniphila*, but an enriched abundance of *Bacteroides fragilis* and *Bacteroides ovatus*. Moreover, depletion of *Akkermansia muciniphila, Faecalibacterium prausnitzii*, and *Bacteroides uniforms* have been reported in IBD ^15^ and could account for the inappropriate immune responses in the host since these are important commensals in the gut. Another study reported that the most significantly elevated species in CD compared with controls were *Ruminococcus gnavus* and *Eggerthella lenta* whereas *Faecalibacterium prausnitzii* and *Eubacteria (E. eligens and E. rectale)* species were decreased. In UC, *E. lenta, Clostridium innocuum*, and *Holdemania filiformis* were increased, while *E. eligens* and *Clostridium aminobutyricum* were decrease relative to healthy controls.^84^ The sources of samples, analytical techniques, levels of microbiota changes, and differential potential as elaborated in this manuscript are summarized in Figure 4.

## Discriminatory potentials of gut bacteria

### IBD versus healthy subjects

Several studies have demonstrated that IBD cases can be accurately predicted and differentiated from healthy controls using gut microbial profiles with the area under the receiver operating characteristic curve (AUROC) ranging from 0.83 to 0.92 based on several machine learning algorithms in many studies.^11,15,17,18,84,85^ There have already been attempts to develop diagnostic tools for IBD and other dysbiotic gut conditions by using fecal samples via profiling intestinal bacteria to identify and characterize dysbiosis. In one of such studies, the authors employed fifty-four DNA probes that target ≥300 bacteria on different taxonomic levels, which were capable of distinguishing between a healthy gut and a dysbiotic gut. Validation analysis confirmed dysbiosis in 70% of treatment-naïve IBD patients, 80% of IBD patients in remission, and 73% of IBS patients compared to 16% in healthy individuals.^14^ In the same study, the predominant bacteria contributing to dysbiosis within the IBD cohort were Proteobacteria (*Shigella/Escherichia*), Bacteroidetes (Bacteroides and Prevotella), and Firmicutes, specifically *Faecalibacterium prausnitzii*. Although this technique needs further improvement, it represents a promising possibility of utilizing the gut microbiota as a diagnostic strategy. Decreased abundance of obligate anaerobes and butyrate producers such as *Faecalibacterium prausnitzii* and *Roseburia hominis* and the enrichment of facultative anaerobes such as *E. coli* characterize the taxonomic perturbation during dysbiosis in IBD. Moreover, increased *Ruminococcus gnavus* and *Ruminococcus torques*, two prominent species in IBD (dysbiotic UC and CD), differentiate IBD from healthy individuals.^86^ Similarly, IBD samples were found to display significantly lower abundances of putative beneficial OTUs present in healthy individuals, including *Prevotella copri* and *Faecalibacterium prauznitzii*, where the abundance of Enterobacteriaceae correlates with ileal CD and Ruminococcaceae correlates with a healthy gut.^87^ It is also asserted that the main genera contributing to the increased abundance of Proteobacteria and Actinobacteria in IBD are *Escherichia, Haemophilus, Bifidobacterium*, and *Collinsella*.^79^ Gevers and colleagues detected positive correlations between CD and the abundances of Pasteurellaceae (*Haemophilus sp*.), Neisseriaceae, Veillonellaceae, and Fusobacteriaceae. Interestingly, *Fusobacterium* such as *Fusobacterium nucleatum* has previously been suggested as a biomarker for IBD,^88^ a marker for the early gut microbial dysbiosis,^89^ and has been shown to promote a beneficial microenvironment for the progression of colorectal carcinoma,^90^ a long-term complication of IBD; and even predominantly associated with cancer cells in metastatic lesions.^91^

Machiels and colleagues found a reduction in *Roseburia hominis* and *Faecalibacterium prausnitzii*, both well-known butyrate-producing bacteria of the phylum Firmicutes in UC patients compared to healthy controls.^92^ In a similar study, *Faecalibacterium prausnitzii*, also a known beneficial bacterium with anti-inflammatory properties was found to be lower in individuals with CD compared with healthy controls.^11^ In addition to *Faecalibacterium prausnitzii*, bacteria species positively influencing gut homeostasis such as *Eubacterium rectale* are significantly decreased in abundance in IBD whereas known pathobionts like *Escherichia coli* are enriched.^93^ Moreover, Firmicutes genera belonging to the order Clostridiales (both the Lachnospiraceae and Clostridiaceae families) such as *Faecalibacterium, Ruminococcus, Eubacterium*, and *Roseburia* are found to be consistently lower in both UC and CD patients than in healthy controls, whereas the abundance of *Lactobacillus* and *Streptococcus* belonging to the order Lactobacillales is increased.^79^ These observations suggest that harnessing species-specific reductions in the abundance in the members of the phylum Firmicute, as well as increases in other phyla like Proteobacteria, may produce a sensitive diagnostic tool for IBD and even differentiate the subtypes. This is consistent with an earlier study that revealed significant differences between the gut microbiota of IBD patients and those of non-IBD controls, with results indicating that a subset of CD and UC samples contained abnormal gastrointestinal microbiota characterized by depletion of commensal bacteria, notably members of the phyla Firmicutes and Bacteroidetes but increase in Proteobacteria.^32^ In a population-based case-control study, the analysis of 84 biopsy specimens revealed a higher abundance of *E. coli* belonging to the B2 + D phylogenetic group in patients with UC and CD than with controls.^94^ Interestingly, an earlier study reported that *E. coli* of the phylogenetic group B2 were isolated more frequently from IBD patients with present or past involvement of the left side of the colon compared to healthy controls.^95^ These observations are highly promising and require further integration of these specific species in a single platform to validate their sensitivity and specificity in IBD diagnosis. Moreover, given the complex nature of the gut microbiota with variations even among healthy individuals, more large cohort studies are needed to establish a sensitive and specific pool of bacteria that characterize the dysbiosis in IBD.

### CD versus UC

Patients with CD and UC show similar dysbiotic gut microbiome profiles, although CD has been noted to be more dysbiotic than UC compared to healthy individuals.^85^ Detailed analysis of the bacteria composition could reveal distinctive features between the two subtypes of IBD. For example, a study found that of the 102 UC-associated bacterial taxa identified, 87 were also found to be associated with the gut microbiome profiles of patients with CD, nonetheless, 15 of the bacterial taxa were UC-specific, including the species *Bacteroides uniformis* and *Bifidobacterium bifidum*.^11^ A similar study found that although a profile *of Faecalibacterium prausnitzii, Ruminococcus gnavus*, and *Bifidobacterium adolescentis* served as a CD-dysbiosis signature and significantly differentiated it from healthy controls, it failed to differentiate between UC patients and controls,^92^ suggesting that different species drive dysbiosis in CD and UC. In treatment naïve patients, the general bacteria community abundance is significantly higher in CD compared with UC for the class Bacilli and the order Lactobacillales by 8-fold, family Streptococcaceae by 5-fold, and genus Desulfovibrio by 13-fold; and lower for the class Clostridia and the order Clostridiales by 1-fold and family Ruminococcaceae by 1-fold.^96^ These observations have led to the constitution of bacterial profiles that not only distinguish UC and CD from healthy persons but also UC from CD. A microbial signature for CD that employed a Boolean algorithm on eight differentially abundant taxa, produced prediction accuracy of 64%–82% and 77%–85% to distinguish CD patients from UC and healthy controls, respectively.^97^ A similar approach using the Machine Learning technique Extreme Gradient Boosting for heterogeneous datasets produced 84% accuracy for CD versus control, 83% accuracy for UC versus control, and 64% accuracy for CD versus UC, with *E. rectale* and *Clostridium cluster XIVa* being the most important discriminatory OTUs in both diseases, respectively. Further analysis resulted in higher predictive accuracies of AUCs of 0.81 (81% accuracy; *Hydrogenoanaerobacterium saccharovorans* most important) for CD, 0.73 (85%; *Bifidobacterium*) for UC and 0.91 (89% accuracy; *Anaerostipes hadrus*) for patients combined.^84^

**Figure 5.**
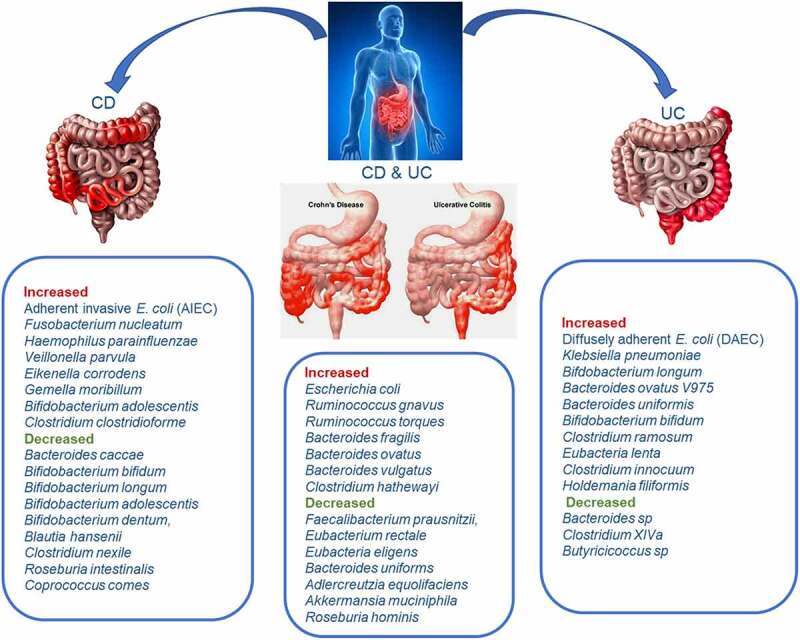
The differential potential of gut bacteria for CD and UC at the species-level. The integration of species level information regarding increases and decreases of specific key dysbiosis-associated bacteria could lead to accurate differentiation of IBD patients from healthy individuals and between the subtypes of IBD (CD from UC). This figure summarizes reported bacteria species increases and decreases in CD, UC, and both types.

According to a meta-analysis by Walters and colleagues, there are increases in *Bifidobacterium adolescentis* and *Lactobacillus* in colonic CD and *Lactobacillus* in ileal CD but not UC as compared to healthy controls.^15^ It is also documented that in addition to the decrease in *Faecalibacterium prausnitzii* in CD, there is also an increase in *Clostridium clostridioforme, Ruminococcus gnavus*, and *Escherichia coli* compared to UC.^85^ On the other hand, a recent study found UC samples to be characterized by an increased abundance of *Escherichia coli, Klebsiella pneumoniae, Bifdobacterium longum* subsp. Longum, and *Bacteroides ovatus V975*, but decreased uncultured Bacteroides sp.^43^ Other studies indicate that UC samples are characterized by a reduced bacteria profile of *Eubacterium rectale, Roseburia hominis, Clostridium XIVa, Butyricicoccus*, and *Faecalibacterium prausnitzii* of the Ruminococcaceae and Lachnospiraceae families, but increases in *Ruminococcus gnavus, Clostridium ramosum* and *E. coli* ^92,93,98^ Moreover, studies of both animal models and cell cultures reveal pathogenic features of *E. coli* pathobionts which likely links them to IBD pathogenesis. Differentially, diffusely adherent *E. coli* (DAEC) has been linked with UC whereas adherent invasive *E. coli* (AIEC) has been associated with CD patients.^99^ This means that specific bacteria profiles may be associated with and define dysbiosis in patients with the different subtypes of IBD, and sufficient species-level data may help differentiate not only these subtypes from the healthy individual but also between the subtypes (Figure 5). However, high-quality evidence is still needed in future explorations,

### Active/relapse disease versus remission

The degree of intestinal dysbiosis in IBD patients correlates with disease activity and severity. In UC patients with active disease and CD patients with aggressive disease, the gut microbiota richness (the number of OTUs) and diversity are significantly decreased, along with a reduction in Firmicutes and elevation in Proteobacteria compared to the inactive disease.^83^ However, according to Serrano-Gómez and colleagues, neither microbiota richness (α- and β-diversity) nor dysbiosis scores successfully differentiate disease activity status (relapse vs. remission) for either CD or UC, but instead, alterations at the species and metabolic pathway levels. Relapse CD samples were enriched in three bacterial species (*Ruminococcus torques, Clostridium bolteae, Fusicatenibacter saccharivorans*) but depleted of 10 species compared to remission samples, including *Bifidobacterium pseudocatenulatum, Bifidobacterium adolescentis, Eubacterium eligens, Streptococcus salivarius, Streptococcus parasanguinis, Blautia hansenii, Intestinibacter bartlettii*, and *Proteobacteria bacterium CAG 139*.^85^ A predictive profile for differentiating active CD versus remission, constructed from these bacteria alterations yielded an AUC of 0.769 on a validation cohort.^85^ Tthe enriched species in the relapse patients, *Ruminococcus Torques* and *Clostridium bolteae*, have also been confirmed in fecal samples of patients with autism spectrum disorder and neuromyelitis optica spectrum disorders, respectively,^100,101^ where *Clostridium bolteae* correlated with the level of inflammatory genes linked with both adaptive immunity and innate immunity and particularly promotes plasma cell differentiation, Th17 activation, and B cell chemotaxis.^101^ By comparing the number of B2 strains *E. coli* having at least one positive extraintestinal pathogenic *E. coli* (ExPEC) gene among study subjects, researchers found that 86% were positive among active IBD patients compared with 13% among inactive IBD patients, and 11% among healthy controls (p < 0.05).^95^ These findings offer the prospect that deeper analysis of large gut microbiota data could reveal distinctive microbial profiles for differentiating between active or relapse and remission IBD patients.

Other studies have reported the relationship between gut bacteria and disease activity/behavior or phenotypes. For example, Bacilli, represented by *Streptococcus*, are significantly increased in patients with mild CD whereas severe CD patients exhibit increased Proteobacteria and Enterococcaceae and decreased Ruminococcaceae and Clostridiales. Mild UC patients showed elevated levels of Bacteroidia and Pseudomonadaceae, moderate UC showed elevated *Streptococcus*, and severe UC exhibited enriched Proteobacteria and Bacilli.^102^ In addition, while the phylum Proteobacteria are enriched in penetrating CD, Pseudomonadaceae and Enterobacteriaceae are rather enriched in both fistulizing CD and stricturing CD. The levels of Clostridiales were reduced in all active UC patients and the elevated abundance of Proteobacteria (Enterobacteriaceae) also markedly correlates with CD severities.^102^

### IBD versus other gastrointestinal diseases

#### IBD versus IBS

Disease-specific associations such as an increased abundance of *Bacteroides* species, including *Bacteroides fragilis* and *Bacteroides vulgatus* are reported to only increase in patients with CD or UC but not IBS.^11^ Bacteroides are typically symbionts but can also be opportunistic pathogens. Interestingly, *Bacteroides fragilis* is previously linked to impaired bacterial tolerance handling by CD-associated genetic variation in the genes ATG16L1 and NOD2, while *Bacteroides vulgatus* is associated with CD pathogenesis and NOD2 host genetic variants.^11,103,104^ Moreover, an increase in species of the Enterobacteriaceae family, including *Escherichia/Shigella* species was observed only in patients with CD. These species are known to invade the gut mucosal epithelium and cause bloody diarrhea, and ulceration of the colon, which are also typical complications of UC.^11,105^ A microbial profile composition generated through machine learning showed a better prediction accuracy (AUC)_mean_ = 0.91 (0.81 to 0.99)] than fecal inflammation biomarker calprotectin AUC_mean_ = 0.80 (0.71 to 0.88) in predicting IBD from IBS. Moreover, there was a distinct exhibit of bacteria growth dynamics, expression of metagenomics-associated functional changes, and even violence factors in the microbiota between IBD and IBS,^11^ indicating a gut microbiota composition and functional potential that could be useful in the deployment of new diagnosis tools in clinical practice. However, Casén and colleagues found that Proteobacteria (*Shigella/Escherichia*) was among the top five dysbiosis‐contributing bacterial groups for both IBS and IBD, along with increased *Actinobacteria* and *Ruminococcus gnavus* in IBS and decreased *Faecalibacterium prausnitzii* and Bacteroidetes (*Bacteroides* and *Prevotella*) in IBD.^14^ The apparent discrepancies confirm the expected geographical deviations in microbial patterns across different countries and the similarities in the underlying dysbiosis in both IBS and IBD. Nonetheless, there are distinct profiles of predominant bacteria contributing to dysbiosis within each cohort and thus, further exploration is needed.

#### IBD versus CRC

**Figure 6.**
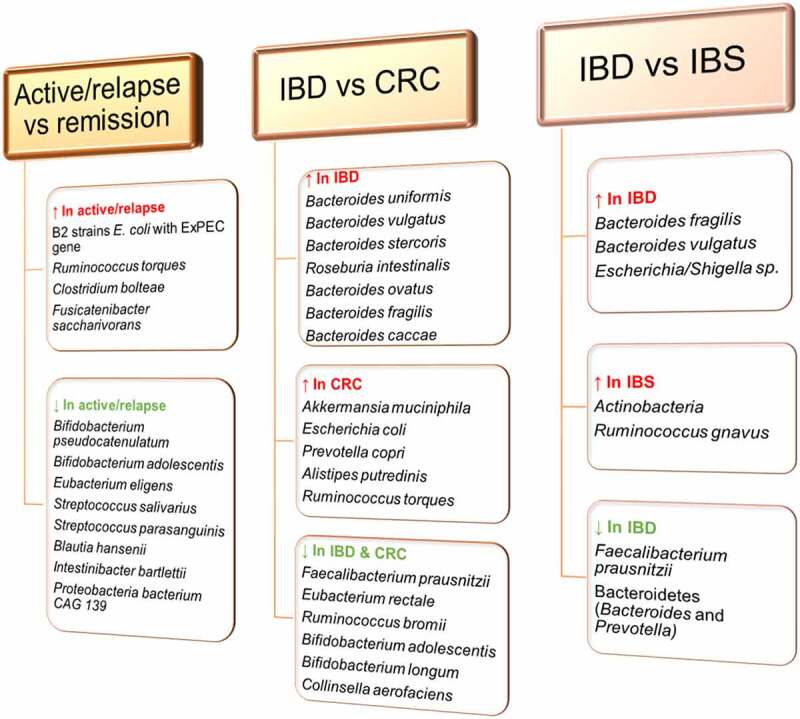
Differential potential of gut bacteria at species-level. Specific bacteria species-changes drive the dysbiosis associated with IBD and other gastrointestinal diseases. These increases and decreases can be harnessed to differentiate an active or relapsed IBD from remission, and IBD from CRC and IBS. However, large studies are required to confirm these observations and expand the available data.

Whereas IBD and CRC share certain specific dysbiotic profiles, including increases in *Escherichia coli* and Enterotoxigenic *Bacteroides fragilis, Fusobacterium nucleatum* appears to be more associated with CRC.^106–108^ An earlier study found that *Fusobacterium nucleatum* does not trigger colitis and exacerbate colon inflammation in APC (Min/+) mice, nor aggravate intestinal inflammation and induce tumors in colitis models of IL-10(-/-) and T-bet(-/-)/Rag2(-/-) mice.^109^ This implies that the tumorigenesis of *Fusobacteria*-mediated tumor progression may be less associated with inflammation. Several studies have implicated *Fusobacterium nucleatum* as a risk factor in the initiation and progression of CRC.^110,111^ A metagenome-wide association study on the fecal samples of 512 IBD patients, 285 CRC patients, and 290 healthy subjects revealed that although IBD and CRC shared 351 common bacteria species, there were still 122 IBD-specific and 60 CRC-specific bacterial species. Increased abundance of *Bacteroides* in IBD differentiated it from CRC patients and healthy controls, whereas *Fusobacteria* differentiated CRC from IBD and healthy controls. At the species level, differential bacteria included an increased abundance of *Bacteroides uniformis, Bacteroides vulgatus, Bacteroides stercoris, Roseburia intestinalis, Bacteroides ovatus, Bacteroides fragilis*, and *Bacteroides caccae* in IBD patients and higher enrichment of *Akkermansia muciniphila, Escherichia coli, Prevotella copri, Alistipes putredinis*, and *Ruminococcus torques* in CRC patients.^70^ This was coupled with reduced abundance of beneficial gut bacteria such as *Faecalibacterium prausnitzii, Eubacterium rectale, Ruminococcus bromii, Bifidobacterium adolescentis, Bifidobacterium longum*, and *Collinsella aerofaciens* in both IBD and CRC.^70^ This indicates that although Proteobacteria are found to increase in both IBD and CRC, they turn to be higher in CRC, along with other phyla such as Fusobacteria and Verrucomicrobia. The species-level information for active versus remission, IBD versus CRC, and IBD versus IBS is presented in Figure 6.

#### IBD versus celiac disease (CeD)

Reports from fecal samples and duodenal biopsies indicate an increased abundance of Gram-negative bacteria such as *Bacteroides, Clostridium*, and *E.Coli* in CeD patients.^112,113^ *E. coli* strains with similar genetic backgrounds and virulence gene profiles have been implicated in the pathogenesis of intestinal disorders such as UC, CD, colorectal cancer (CRC), and celiac disease (CeD). A recent study found that all AIEC strains isolated from UC and CRC belonged to the B1 phylogroup (except for a strain of the A phylogroup), compared to the CeD cohort which had none of the isolated *E. coli* being AIEC.^114^ This implicates the integration of strain-specific microbial profiles in differentiating IBD from other gastrointestinal conditions. Other reports reveal that *Clostridium spp., Prevotella spp*., and *Actinomyces spp*. characterize the dysbiosis in the small bowel mucosa of CeD patients.^115,116^ Although a number of other studies have outlined the dysbiotic profile of CeD samples,^117,118^ there is a lack of data on a comprehensive differential analysis of significant gut bacteria between IBD and CeD. Future studies are therefore needed as they could reveal distinct diagnostic profiles.

## Prognostic potentials

The increased or decreased abundance of certain gut bacteria and the overexpression of microbial-associated genes and toxins have been noted to correlate with IBD prognosis. A higher relative abundance of 142 genes encoding antibiotic resistance proteins was found in CD compared with controls, out of which 63 were components of efflux complexes that remove antibiotics from bacteria, thus impeding the effectiveness of antibiotics. Further analysis indicated that the antibiotic resistance protein TolC was upregulated in patients with CD and correlated with taxonomy abundance of the genus *Escherichia*, which was also increased. Moreover, patients with UC had upregulated levels of 66 antibiotic resistance proteins, including cepA, a β-lactamase enzyme that mediates resistance to β-lactam antibiotics,^11,119^ and the elevated cepA correlated with the abundance of the genus *Bacteroides*, which was increased in both CD and UC.^11^ Similarly, a shift toward antibiotic-resistant taxa in CD and UC groups distinguished them from healthy controls, in addition to *E. coli* abundance correlating positively with several virulence genes.^93^ Bacteriological analyses of biopsy specimens from IBD patients show elevated numbers of *E. coli* isolates belonging to the B2 phylogenetic group that harbors extraintestinal pathogenic *E. coli* (ExPEC) genes,^94^ and virulence factor determinants encoding fimbriae (fimA), cytotoxic necrotizing factor (cnf1), aerobactin synthesis (aer), and the locus associated with invasiveness (ial) are more prevalent in IBD *E. coli*.^120^ This presents the expansion of B2 phylogenetic *E. coli* in IBD as a prognostic marker for poor patients’ outcome. CD is associated with gut inflammation spanning multiple tissue layers, with deep ulceration correlating with worse long-term disease outcomes. A group of researchers measured the incidence of deep ulceration in CD patients during a diagnostic colonoscopy, allowing them to assess the association between gut microbiota and mucosal ulceration. They documented a prevalence rate of 42% deep ulceration (colon or ileum) in CD patients and noticed an elevated abundance of Pasteurellacaea, Veillonellaceae, and *Rothia mucilaginosa*. Moreover, an association between the KEGG pathway for pathogenic *Escherichia coli* infection positively correlated with ulcer formation.^55^ These observations imply that these gut bacteria species may contribute to ulceration in IBD and thus, may serve as markers for long-term disease outcomes. However, further studies are needed to establish whether these bacteria casually participate in ulceration in IBD patients, or are merely adapted to live in this affected environment.

A number of studies have reported microbiome-based prediction profiles for key clinical endpoints of IBD such as response to treatment and post-operative recurrence. In a model that produced 87.5% and 79.1% prediction accuracy in CD and UC patients, respectively, in combined Western and Chinese IBD cohorts, there was a strong correlation between IBD severity and a relative increase in the abundance of Actinobacteria and Proteobacteria (Enterobacteriaceae) and decrease in Firmicutes (Clostridiales). Further analysis indicated that while the restoration of gut microbiota diversity and increase in Clostridiales abundance was associated with patients’ response to infliximab treatment, certain bacteria groups, mainly Clostridiales, predicted 86.5% accuracy of treatment effectiveness alone or 93.8% accuracy in combination with calprotectin levels and Crohn’s disease activity index (CDAI).^102^ Another study found that baseline microbial richness indicates preferential responses to anti-cytokine (anti-TNF or -IL12/23) therapy and correlates with the abundance of microbial species capable of 7α/β-dehydroxylation of primary to secondary bile acids. The abundance of microbial species of the Firmicutes phyla including *Faecalibacterium prausnitzii* and *Ruminococcus bromii* were associated with increased clinical remission at week 14 (73.3% versus 41.2%), week 52 (66.7% versus 36.4%), and endoscopic remission (65% versus 37.5%,) compared with the abundance of *Bacteroides ovatus, B. thetaiotaomicron*, and *Veillonella parvula*. The researchers found unique remission-associated multi-omic profiles with each therapeutic class, providing a potential prior determination of optimal therapeutics for patients and serving as targets for newer therapies.^121^ Moreover, non-responders to Infliximab treatment have lower abundances of bacteria that produce short-chain fatty acids, particularly of the class Clostridia, and higher levels of pro-inflammatory bacteria and fungi, such as the genus Candida, compared with responders.^96^ Compared with the postoperative non-recurrence setting in IBD, endoscopic recurrence is linked with marked alteration in ileal mucosa-associated microbiota, including reduced alpha diversity and Firmicutes (family Lachnospiraceae and Ruminococcaceae) such as genera *Ruminococcus, Eubacterium, Butyricoccus, Dorea*, and *Blautia*, and increased Proteobacteria (family Alphaproteobacteria and Coribacteriaceae and genus *Enterococcus*). In addition, gut bacteria at surgery that potentially predict postoperative endoscopic recurrence include Gammaproteobacteria, *Ruminiclostridium 6*, and *Ruminococcus gnavus group*.Other.^122^In a similar study, mucosal enrichment of Lachnospiraceae and Fusobacteri-aceae and decrease of Streptococcaceae and Actinomycineae served as a predictor of postoperative CD recurrence, with six months post-operative assessment indicating a higher abundance Negativicutes and Fusobacteria in recurrence patients compared to non-recurrence.^123^ These observations present the gut microbiota as a potential prognostic marker of IBD post-operative recurrence and drug response, with the specific bacterial profiles also serving as therapeutic targets, however, more large studies and detailed data mining are required.

There is an inverse correlation between the abundance of *Faecalibacterium prausnitzii* and disease activity in UC as the bacterial count was found to be lowest in UC patients with severe disease compared to moderate and quiescent disease. The same trend was observed with *Roseburia hominis*.^92^ Some of the mechanisms by which *Faecalibacterium prausnitzii* abrogates gut inflammation are the production of microbial anti-inflammatory molecules,^124^ regulation of intestinal integrity, and production of butyrate to maintain Th17/Treg balance.^125^ *Roseburia hominis* protects against colitis by enhancing gut barrier function, symbiont host colonization, upregulating genes related to antimicrobial peptides, and Treg population expansion, possibly via TLR5-flagellin signaling.^126^ Interestingly, ileocecal resection causing the removal of the ileocecal valve in patients with CD served as a key factor associated with a reduced gut microbial and gene richness, including a reduced relative abundance of beneficial bacteria such as *Faecalibacterium prausnitzii* and the Ruminococcaceae family, but an elevated abundance of *Fusobacterium*.^11^ This indicates that the removal of the ileocecal valve has a negative consequence on the gut microbiota of patients with IBD. The potent anti-inflammatory effects of these species explain why their count is lowest in severe IBD compared to moderate and quiescent diseases. On the other hand, increased Escherichia-Shigella and Enterococcus positively correlate with inflammatory markers and negatively correlate with nutrition markers, which indicates a more severe disease in pediatric IBD.^127^ The correlation between disease activity and severity could serve as a good prognostic marker and thus, require further investigation since several studies have also reported similar microbial patterns in both CD and UC. Moreover, an axis characterized by an elevated abundance of gut bacteria such as Enterobacteriaceae, Pasteurellacaea, Veillonellaceae, and Fusobacteria-ceae, and reduced abundance of Erysipelotrichales, Bacteroidales, and Clostridiales, strongly correlated with disease status in new-onset CD patients.^55^ Therefore, deeper mining of these data could produce effective profiles capable of differentiating disease status and severity. Table 1 summarizes the predictive potential of gut bacteria as discussed above.

**Table 1.** Diagnostic ability of gut bacteria in IBD clinical studies.

Sample source	Study type/focus	Study subject	Analytical technology	Bacteria involved	Key findings	Reference
Biopsy	Population-based case-control	CD and UC	RISA followed by DNA sequencing	B2 + D phylogenetic *E. coli*	More prevalent in patients with UC and CD than controls	^94^
Biopsy and fecal	Prevalence of AIEC in selected cohorts	UC, CRC, CeD	Culture and identification	*E. coli*	No AIEC found in CeD. AIEC prevalence higher in CD: 55.0% than CD with colonic disease: 40.0% and UC: 35.7%	^114^
Fecal	Characterizing isolated *E. coli* strains	IBD and healthy controls	Culture and identification	*E. coli* strains	Phylogenetic group B2 *E. coli* are frequent in IBD involving the left side of the colon. B2 strains with ExPEC genes are more frequent in active IBD than inactive IBD	^95^
Fecal	Case-control analysis	UC, CD, IBS UC and healthy controls	Shotgun metagenomic sequencing	*Bacteroides uniformis* and *Bifidobacterium bifidum* *Faecalibacterium prausnitzii* *Escherichia/Shigella* species	15 UC-specific associations, including *Bacteroides uniformis* and *Bifidobacterium bifidum* Decreased in UC compared to controls Increased in CD	^11^
Fecal	Cross-sectional	IBD and healthy controls	16S rRNA gene sequencing	Proteobacteria (*Shigella/Escherichia*), Firmicutes, specifically *F. prausnitzii*, and Bacteroidetes (*Bacteroides and Prevotella*)	Diagnostic rate of 70% in treatment‐naïve IBD patients and 80% in IBD patients in remission, with 16% false positivity in healthy individuals	^14^
Biopsy	Characterizing mucosa-associated bacteria	IBD and healthy controls	16S rRNA gene sequencing and bacteria culture	*Akkermansia muciniphila*, *Ruminococcus torques*, and *Ruminococcus gnavus*	A. muciniphilia, was reduced many folds in IBD but R. *torques* and *R. gnavus* increased by ∼100 and >4-fold respectively	^16^
Fecal	Retrospective metagenomics data analysis	IBD and CRC	Metagenomics dataset retrieval	*Bacteroides*, including *Bacteroides uniformi, Bacteroides vulgatus, Bacteroides stercoris, Roseburia intestinalis, Bacteroides ovatus, Bacteroides fragilis*, and *Bacteroides caccae, and* *Fusobacteria*	Increased *Bacteroides* differentiated IBD from CRC and healthy subjects while *Fusobacteria* differentiated CRC from IBD and controls	^70^
Fecal	Cross sectional	UC, CD, healthy controls	Denaturing gradient gel electrophoresis (DGGE) and qrt-PCR	*R hominis, F prausnitzii, B adolescentis*, and *R gnavus*	R hominis and F prausnitzii differentiated UC from healthy individuals while F prausnitzii, B adolescentis, and R gnavus differentiated CD from healthy control	^92^
-	Systemic review and meta-analysis	UC, CD, healthy controls	16S rRNA gene sequencing	*Bifidobacterium adolescentis* and *Lactobacillus*	Increased in both colonic Crohn’s disease and ileal Crohn’s disease but not UC as compared to healthy controls	^15^
Biopsy	Blinded study comparing virulence potential of isolated *F. nucleatum* strains	IBD, IBS, indeterminate colitis,	Culture and characterization	*Fusobacterium nucleatum*	Inflamed biopsy-associated *F. nucleatum* strains were significantly more invasive. Highly invasive strains of *F. nucleatum* may be a useful biomarker for gastrointestinal disease	^88^
Fecal	Case-controlled	UC, CD, controls	Whole genome shotgun sequencing	*Eubacterium rectale* and *Faecalibacterium prausnitzii*	Significantly reduced in UC but not CD and controls	^93^
Fecal	Cross-sectional	UC, control	16S rRNA and metagenomic shotgun sequencing	*Akkermansiaceae, Clostridiaceae, Eggerthellaceae, Lachnospiraceae*, and *Oscillospiraceae*	Species depletion in these families differentiated pediatric UC from controls. UC could be predicted with an AUROC of about 0.90	^17^
Biopsy and fecal	Multi-cohort characterizing treatment-naïve microbiome	CD, non-IBD controls	16S rRNA gene sequencing	*Escherichia coli, Fusobacterium nucleatum, Haemophilus parainfluenzae (Pasteurellaceae), Veillonella parvula, Eikenella corrodens (Neisseriaceae), Gemella moribillum* *Bacteroides vulgatus, Bacteroides caccae, Bifidobacterium bifidum, Bifidobacterium longum, Bifidobacterium adolescentis, Bifidobacterium dentum, Blautia hansenii, Ruminococcus gnavus, Clostridium nexile, Faecalibacterium prausnitzii, Ruminoccus torques, Clostridium bolteae,*	Increased levels of these species were significantly differential for UC Decreased levels of these species were significantly differential for UC	^55^
Fecal	Cross-section	CD, UC, healthy controls	16S rRNA and whole-genome shotgun sequencing	*Escherichia, Haemophilus, Bifidobacterium*, and *Collinsella* *Faecalibacterium, Eubacterium, Ruminococcus*, and *Roseburia*	Elevated abundance significantly differential for IBD Decreased abundance significantly differential for IBD	^79^
Fecal	Longitudinal intercontinental study	CD, UC, healthy controls	16S rRNA gene sequencing	A microbial signature including *E. rectale* and *Clostridium cluster XIVa* as the most important discriminators for CD and UC, respectively	Prediction accuracy of 84% for CD versus control, 83% for UC versus control, and 64% for CD versus UC.	^84^
Fecal	Cross-populational (Western and Chinese) IBD cohorts	CD, UC, healthy controls	16S rRNA gene sequencing	*Streptococcus* increased in patients with mild CD. Increased Proteobacteria and Enterococcaceae and decreased Ruminococcaceae and Clostridiales in moderate to severe CD. Increased Bacteroidia, and Pseudomonadaceae in mild UC. Increased *Streptococcus* in moderate UC. Increased Proteobacteria and Bacilli in severe UC	A prediction model based on gut microbiome for IBD diagnosis was robust across the cohorts and showed 87.5% and 79.1% prediction accuracy in CD and UC patients, respectively.	^102^

RISA, Ribosomal intergenic spacer analysis; UC, ulcerative colitis; CD, Crohn’s disease; CRC, colorectal cancer; CeD, celiac disease; ExPEC, extraintestinal pathogenic E. coli; IBS, irritable bowel syndrome;

## Challenges and perspectives

Due to the substantial overlap in the clinical presentation of IBD and other gastrointestinal diseases, it could be difficult for a gastroenterologist or general practitioner to differentiate between these conditions, thus, colonoscopies are performed on a large number of patients to reach an accurate diagnosis. However, colonoscopy presents several risks such as bleeding, perforated intestine, post-polypectomy electrocoagulation syndrome, and adverse reaction to anesthetic.^128,129^ Fortunately, fecal matter presents a convenient and repeatable sampling that is noninvasive, inexpensive, and offers sufficient biomass for gut microbial analysis, which has been demonstrated to provide promising diagnostic and prognostic profiles for IBD. Notwithstanding, some studies report that fecal samples present a bacteria profile more similar to that of healthy controls compared with musical biopsies.^130^ Regardless, most available studies employed fecal samples, partly due to the convenient sampling process. With the assertion that microbiota adhering to the gut mucosa better discriminates IBD patients from controls, a convenient sampling procedure should not be the only driving factor in obtaining microbiota samples for diagnosis. Perhaps, more data from mucosa biopsies or its integration with fecal samples could produce the needed bacteria profile for differentiating between the types of IBD and IBD from other gastrointestinal diseases.

Cross-sectional studies give an overview of the relative abundance of bacterial taxa at a single time point and therefore do not capture the complex dynamics of the microbial ecosystems in the gut of patients with IBD. Moreover, geographical deviations in microbial patterns exist across IBD patients from different countries. As a way of overcoming this challenge, studies have demonstrated that bacterial growth dynamics could be inferred from a single metagenomic sample by analyzing the pattern of sequencing read coverage across gut bacteria genomes.^131^ This may provide a standardized approach to the assessment of IBD-associated bacterial growth that could be replicated in different geographical locations. The assessment of disease-associated growth rate differences could assist in identifying actively growing bacteria, hence, helping to prioritize disease-associated taxonomy results. For example, bacterial growth rates were determined for 40 species out of which four species and five species were altered in patients with CD and UC respectively, compared with control individuals.^11^ Given the richness and the significant number of different bacteria species per person per sample of gut microbiota, developing and improving techniques that highlight disease-associated bacteria taxonomies may lead to highly sensitive and specific diagnostic markers for IBD.

The observed variable gut bacteria changes across various studies could likely reflect the lack of standardization across existing microbiome techniques, or perhaps the result of the heterogeneity in the microbiome associated with the disease. It is reported that geographic location accounts for most of the microbiota variance, second to the presence or absence of CD, followed by patients’ history of surgical resection, alcohol consumption and UC diagnosis, medications and diet with most (90.3%) of the compositional variance stochastic or unexplained.^84^ This presents a unique challenge to identifying highly specific and sensitive microbiome-based biomarkers for IBD. Again, different microbiome technologies are likely to vary in results. For example, a study that used both 16S rRNA gene sequencing and metagenomic shotgun sequencing documented significant differences in UC-associated species by the different techniques. Only the shotgun techniques but not 16S rRNA detected significant decreases in species such as *Bifidobacterium bifidum, Adlercreutzia equolifaciens, [Eubacterium] rectale*, etc. and increases in *Escherichia coli, Klebsiella pneumoniae Bifidobacterium breve, Bacteroides fragilis, Enterococcus faecalis, Lactobacillus gasseri, Lactococcus lactis, Dialister pneumosintes, Morganella morganii, Porphyromonas asaccharolytica*, etc.^17^ Similar observations were made in the 16S rRNA gene sequencing techniques as it detected certain UC-associated species not found in the shotgun analysis. This implies that standardization is needed not only within a given technique but across different techniques to ensure consistency and reproducibility of results. Moreover, IBD treatments potentially affect the composition and diversity of the gut microbiota as indicated in studies such as that of Zuo and colleagues,^17^ who found that after the exclusion of UC cases undergoing treatment, species from the Sutterellaceae, Peptoniphilaceae, and Erysipelatoclostridiaceae families were no longer associated with UC contrary to their significant involvement in overall UC cases using 16S rRNA data. On the contrary, species from Firmicutes RF39 and Hungateiclostridiaceae were found to be reduced in treatment-naïve UC cases. This means that distinct microbial profiles characterize not only IBD types but subtypes. Thus, studies should consider profiling gut bacteria according to IBD subtypes since data on large cohorts is lacking.

Although recent advances on the differential potential of gut bacteria are encouraging, there is the need to integrate more species‐level information into gut microbiota diagnostic profiles and target a wide range of variable positions in the 16S rRNA gene, which could lead to the development of a low error rate diagnostic tool. For example, by using different training datasets of IBD microbiota, the authors observed that species-level data performed best for almost all comparisons between healthy controls and individual disease states, and all disease states combined, by accurately differentiating UC versus healthy controls (ROC AUC = 0.92), ileal CD versus healthy controls (ROC AUC = 0.97), colonic CD versus healthy controls (ROC AUC = 0.88), and all IBD categories versus healthy controls (ROC AUC = 0.92).^15^ Thus, judicious use of supervised learning that is optimized to maximize sensitivity and specificity, can be used to differentiate IBD versus healthy individuals and IBD subtypes, with a significant degree of accuracy when bacteria species are employed.

Finally, data on other ‘omics such as metabolomics and microorganisms like fungi and viruses, which have also shown diagnostic or predictive ability, could be integrated with bacterial profiles to enhance sensitivity and specificity. For example, a recent study found that although neither metagenomic nor host genetics alone could distinguish CD location subtypes, a combined multi-omic feature set or mass spectrometry-based metabolomics and metaproteomics could differentiate ileal and colonic CD. The multi-omic feature set showed that colonic CD is strongly linked with neutrophil-related proteins and exhibits a disease-severity-related association with *Bacteroides vulgatus*. In addition, ileal CD profiles exhibit elevated levels of primary and secondary bile acid and accompanying changes in taxa, particularly *Faecalibacterium prausnitzii* or affinities for bile acid-rich microenvironments, including *Blautia sp* and *Gammaproteobacteria* compared to colonic CD.^132^ These observations demonstrate the power of multi-omics approaches for IBD diagnostic and prognosis biomarker discovery, providing a vital future research direction. In other studies, IBD patients have been shown to have altered fungal microbiota with disease-specific alterations in diversity, where a CD-specific gut environment may favor fungi at the expense of bacteria. More importantly, the combined analysis of fungal and bacterial microbiota showed a dense and homogenous correlation network in healthy subjects but a dramatically unbalanced network in IBD patients, suggesting the existence of disease-specific inter-kingdom alterations.^38^Further exploration of these connections to constitute a multi-omic profile may produce a breakthrough in IBD biomarker discovery.

## Conclusion

The introduction of 16S rRNA and shotgun metagenomic sequencing techniques has allows the exploration of the complexity of the gut microbial ecosystem with high resolution. IBD has a generally consistent microbiome signature across studies and allows high classification accuracy of IBD from non-IBD subjects. More so, distinct bacteria profiles characterize the dysbiosis found in CD and UC, differentiating them from healthy individuals and other gastrointestinal diseases. Current microbiome data is promising and appears to perform better in some studies than the currently used fecal inflammation biomarker calprotectin in predicting IBD from healthy controls and IBS. However, gut bacteria community profiling through next-generation sequencing as a diagnostic tool for IBD is still in the early stage of development and requires more large cohort studies, as well as models that utilize specific disease-associated bacteria at the species level to predict IBD. The quest for rational design of diagnostic, prognostic, or therapeutic manipulation of the microbiota is confronted with not only the heterogeneity of the host response, but also widely different lifestyles, including diet, alcohol consumption, and medications, with much variance still unaccounted for. Moreover, variations in laboratory protocols, sequencing techniques, and geographical origin of samples may influence the microbiome predictive accuracy, thus, the need for standardization. In addition to employing detailed data mining to construct predictive models from key bacterial species, future studies should also target the combination of different fecal or blood biomarkers, including fecal calprotectin, to produce a more sensitive and specific diagnostic tool for IBD.
